# The Health Stigma and Discrimination Framework: a global, crosscutting framework to inform research, intervention development, and policy on health-related stigmas

**DOI:** 10.1186/s12916-019-1271-3

**Published:** 2019-02-15

**Authors:** Anne L. Stangl, Valerie A. Earnshaw, Carmen H. Logie, Wim van Brakel, Leickness C. Simbayi, Iman Barré, John F. Dovidio

**Affiliations:** 1 International Center for Research on Women, 1120 20th St. NW, Suite 500N, Washington, DC, 20036 USA; 2 Department of Human Development and Family Sciences, University of Delaware, Newark, DE USA; 3 Factor-Inwentash Faculty of Social Work, University of Toronto and Women’s College Research Institute, Women’s College Hospital, Toronto, Ontario Canada; 4 Netherlands Leprosy Relief, Amsterdam, Netherlands; 5 Human Sciences Research Council & Department of Psychiatry & Mental Health, University of Cape Town, Cape Town, South Africa; 6 Yale University, New Haven, CT USA

**Keywords:** Stigma, discrimination, health conditions, disease, multi-level, theoretical framework, conceptual model

## Abstract

Stigma is a well-documented barrier to health seeking behavior, engagement in care and adherence to treatment across a range of health conditions globally. In order to halt the stigmatization process and mitigate the harmful consequences of health-related stigma (i.e. stigma associated with health conditions), it is critical to have an explicit theoretical framework to guide intervention development, measurement, research, and policy. Existing stigma frameworks typically focus on one health condition in isolation and often concentrate on the psychological pathways occurring among individuals. This tendency has encouraged a siloed approach to research on health-related stigmas, focusing on individuals, impeding both comparisons across stigmatized conditions and research on innovations to reduce health-related stigma and improve health outcomes. We propose the Health Stigma and Discrimination Framework, which is a global, crosscutting framework based on theory, research, and practice, and demonstrate its application to a range of health conditions, including leprosy, epilepsy, mental health, cancer, HIV, and obesity/overweight. We also discuss how stigma related to race, gender, sexual orientation, class, and occupation intersects with health-related stigmas, and examine how the framework can be used to enhance research, programming, and policy efforts. Research and interventions inspired by a common framework will enable the field to identify similarities and differences in stigma processes across diseases and will amplify our collective ability to respond effectively and at-scale to a major driver of poor health outcomes globally.

## Background

Stigma is a well-documented global barrier to health-seeking behavior [1], engagement in care [2], and adherence to treatment [3] across a range of health conditions [4, 5]. As a distinguished and labelled difference [6], stigma, Goffman notes, enables *varieties of discrimination* that ultimately deny the individual/group *full social acceptance*, reduce the individuals’ opportunities [7], and fuel social inequalities [8]. Stigma influences population health outcomes by worsening, undermining, or impeding a number of processes, including social relationships, resource availability, stress, and psychological and behavioral responses, exacerbating poor health [9].

In order to intervene to halt the stigmatization process or mitigate the harmful consequences of health-related stigma, or stigma associated with health conditions, the existence of a clear, multi-level theoretical framework to guide intervention development, measurement, research, and policy is critical. Existing stigma frameworks typically focus on one health condition in isolation, for example, obesity/overweight [10–17], HIV [8, 18–23], or mental health [24–28]. This tendency has encouraged a siloed approach to research on health-related stigmas, stifling innovative public health responses. Alderson argues that it is practical and scientific to examine theories, as they *powerfully influence how evidence is collected, analysed, understood and used* and notes that, when theories are implicit, their *power to clarify or to confuse, and to reveal or obscure new insights, can work unnoticed* [29]. As such, it is useful to have an explicit theoretical framework that can both guide research and intervention development on individual health conditions and allow for comparisons and responses across health conditions.

The majority of health-related stigma frameworks explore psychological pathways at the individual level, focusing either on the individuals experiencing stigma [10, 11, 14–16, 30, 31], those perpetuating stigma [21, 26], or both [20, 24, 32]. While critical to understanding the factors that facilitate and mediate the stigmatization process for individuals, these frameworks limit researchers’ ability to inform the multi-level interventions required to meaningfully influence the stigmatization process [33]. For some health conditions, including HIV [8, 18, 19, 23, 34, 35], mental health [27, 28], child health [35], and obesity/overweight [17], frameworks addressing the social (e.g. cultural and gender norms) and structural (e.g. legal environment and health policy) pathways leading to stigma, in addition to the individual pathways, have been proposed. A few general stigma frameworks have also highlighted the influence of social and structural forces on the stigmatization process across socio-ecological levels [6, 9, 36]. In the context of health-related stigma reduction, socio-ecological levels have been defined as public policy (national and local laws and policies), organizational (organizations, social institutions, workplaces), community (cultural values, norms, attitudes), interpersonal (family, friends, social networks), and individual (knowledge, attitudes, skills) [37].

Building from existing conceptualizations of health-related stigmas and practical experience in designing stigma-reduction interventions, we propose a new, crosscutting framework and demonstrate its application to a range of health conditions, including leprosy, epilepsy, mental health, cancer, HIV, and obesity/overweight. We discuss how stigma related to race, gender, sexual orientation, class, and occupation intersects with health-related stigmas, and examine how the framework can be used to enhance research, programming, and policy efforts. The framework is intended to amplify our collective ability to respond effectively and at-scale to a major driver of poor health outcomes globally.

## The Health Stigma and Discrimination Framework

**Fig. 1:**
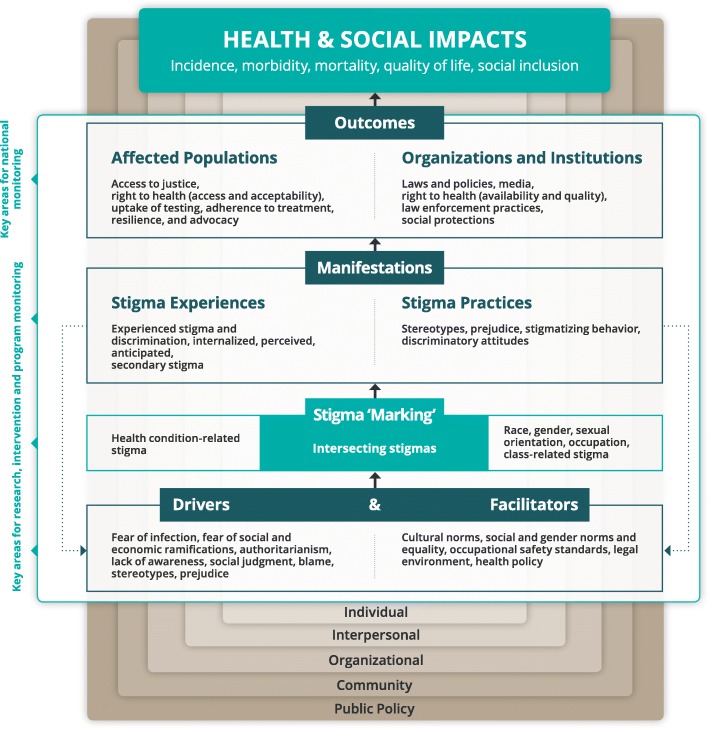
Health Stigma and Discrimination Framework

The Health Stigma and Discrimination Framework (Fig. 1) articulates the stigmatization process as it unfolds across the socio-ecological spectrum in the context of health, which can vary across economic contexts in low-, middle-, and high-income countries. The process can be broken down into a series of constituent domains, including drivers and facilitators, stigma ‘marking’, and stigma manifestations, which influence a range of outcomes among affected populations, as well as organizations and institutions, that ultimately impact health and society.

The first domain refers to factors that drive or facilitate health-related stigma. Drivers vary by health condition, but are conceptualized as inherently negative [18]. They may range from fear of infection through casual contact for communicable diseases and concerns about productivity due to poor health for chronic conditions, to authoritarianism and social judgment and blame. Conversely, facilitators may be positive or negative influences [33], for example, the presence or absence of occupational safety standards and protective supplies in health facilities can minimize or exacerbate stigmatizing avoidance behaviors towards populations with infectious diseases by healthcare workers [38]. Drivers and facilitators determine whether stigma ‘marking’ occurs, through which a stigma is applied to people or groups according to a specific health condition or other perceived difference such as race, class, gender, sexual orientation, or occupation. Intersecting stigma occurs when people are ‘marked’ with multiple stigmas [39]. Once a stigma is applied, it manifests in a range of stigma experiences (i.e. lived realities) and practices (i.e. beliefs, attitudes, and actions). Stigma experiences can include experienced discrimination, which refers to stigmatizing behaviors that fall within the purview of the law in some places, such as refusal of housing [33], and experienced stigma, or stigmatizing behaviors that fall outside the purview of the law such as verbal abuse or gossip [33]. The legal distinction is included as responding to a stigma manifestation that is illegal may require a different response (e.g. litigation) compared with a manifestation that is not illegal. Another stigma experience is internalized or ‘self-stigma’, which is defined as a stigmatized group member’s own adoption of negative societal beliefs and feelings, as well as the social devaluation, associated with their stigmatized status [40]. Perceived stigma (i.e. perceptions about how stigmatized groups are treated in a given context) [41] and anticipated stigma (i.e. expectations of bias being perpetrated by others if their health condition becomes known) are also classified as stigma experiences [42]. Finally, secondary or ‘associative’ stigma, which refers to the experience of stigma by family or friends of members of stigmatized groups or among healthcare providers who provide care to members of stigmatized groups [43], is included under stigma experiences. Stigma practices can include stereotypes (i.e. beliefs about characteristics associated with the group and its members), prejudice (i.e. negative evaluation of the group and its members), stigmatizing behavior (i.e. exclusion from social events, avoidance behaviors, gossip), and discriminatory attitudes (i.e. belief that people with a specific health condition should not be allowed to participate fully in society). We included stereotypes and prejudice under ‘drivers’ and ‘manifestations’, as they both fuel and are reinforced by the stigmatization process.

We postulate that stigma manifestations subsequently influence a number of outcomes for affected populations, including access to justice, access to and acceptability of healthcare services, uptake of testing, adherence to treatment, resilience (i.e. the power to challenge stigma) [34, 44], and advocacy. They also influence outcomes for organizations and institutions, including laws and policies, the availability and quality of health services, law enforcement practices, and social protections.

While the framework is specific to health-related stigma, it recognizes that health-related stigma often co-occurs with other, intersecting stigmas, such as those related to sexual orientation, gender, race, occupation, and poverty. Therefore, incorporating intersecting stigmas into the framework is necessary, as stigma manifestations and health outcomes may be influenced by a range of stigmatizing circumstances that must be considered to understand the full impact of stigma [5, 36].

### How is the framework different?

The Health Stigma and Discrimination Framework differs from many other models in that it does not distinguish the ‘stigmatized’ from the ‘stigmatizer’ [21, 32]. The absence of this dichotomy is intentional, as we seek to challenge the ‘us’ versus ‘them’ distinction that enables people to set others apart as ‘different from the norm’, a key component of the stigmatization process described by Link and Phelan [6], which precedes stigma ‘marking’. As suggested by Parker and Aggleton [8], we seek to move away from psychological models that *see stigma as a thing which individuals impose on others* and instead emphasize, *the broader social, cultural, political and economic forces that structure stigma*.

According to Kippax et al. [45], the danger in separating ‘us’ from ‘them’, or ‘agency’ from ‘vulnerability’, is that it removes the power that vulnerable populations have to act upon the social contexts driving their experiences, behaviors, and actions. The dichotomy also leads to an oversimplified view of vulnerable populations as a group of individuals defined and connected only by the ‘attribute’ of vulnerability [45]. Our framework seeks to show the interconnections between power and vulnerability and how they are fluid and complex. We want to underscore that all individuals can anticipate, perceive, internalize, experience, or perpetuate health-related stigma, while acknowledging unique outcomes for affected populations. There are no clear-cut boundaries about who experiences and who perpetuates stigma, yet, as we highlight throughout each example, stigma intersects with other axes of disempowerment and marginalization (e.g. across race, class, gender) in ways that result in some persons being more disadvantaged by health-related stigma. Removing the ‘us’ versus ‘them’ dichotomy also makes the framework more palatable to change agents, such as community leaders, advocates, and policy-makers, as it highlights that all persons can act as change agents and underscores the need for self-reflection and awareness of biases.

Another difference from previous frameworks is the separation of manifestations into ‘experiences’ and ‘practices’. This distinction clarifies the pathways to various outcomes following the stigma-marking phase of the process. Those who experience, internalize, perceive, or anticipate health-related stigma face a range of possible outcomes, such as delayed treatment, poor adherence to treatment, or intensification of risk behavior, that may diminish their health and wellbeing. While outcomes are mostly negative, positive outcomes are possible; stigma has been known to foster resilience in marginalized populations [46] and fuel the formation of patient advocacy groups and advocacy efforts that have led to major policy changes to improve access to healthcare for some stigmatized conditions like HIV [36, 47]. Stigma practices, on the other hand, highlight how the stigmatization process can generate or reinforce stereotypes and prejudice towards people or groups living with or at risk of various health conditions and foster discriminatory attitudes that fuel social inequalities [8].

We also differentiated outcomes for affected populations (i.e. the stigmatized person or group, as well as their family, friends, or healthcare providers) from outcomes for organizations and institutions. Our framework seeks to demonstrate that stigma experiences and practices influence affected populations as well as organizations and institutions, which then together influence the health and social impacts of stigma. By articulating these outcomes, the framework highlights the need for multilevel interventions to respond to health-related stigma. It also focuses attention on the far-reaching influence of health-related stigma on societies as well as individuals.

### Where to intervene?

Ideally, we want to interrupt the process prior to the application of stigma. Thus, interventions often target the removal of the drivers of stigma or the shifting of norms and policies that facilitate the stigmatization process [33]. However, once a stigma is applied to people with a specific disease or health condition and once it manifests in experiences or practices, interventions are needed to mitigate harm and shift harmful attitudes and behaviors that compromise the general health and wellbeing of affected communities. Stigma-reduction interventions are most effective when they include components directed at a range of actors and socio-ecological levels [37]. A multi-component intervention, for example, may seek to support individuals with leprosy to cope with experienced stigma and overcome internalized stigma, as well as reaching out to community members to shift harmful norms about leprosy through community dialogues or engaging local leaders to share anti-stigma messages [48]. Likewise, advocacy with policy-makers and community leaders about the benefits of syringe exchange programs to prevent transmission of HIV may be combined with training of law enforcement officers on harm reduction and proper implementation of laws that de-criminalize drug use [49].

### What to monitor?

The availability of data on health-related stigma and discrimination is critical for improving interventions and programs to address them, yet such routine data are often lacking [33]. The Health Stigma and Discrimination Framework indicates key areas of focus for program-, facility-, and national-level monitoring. At the program level, data on the drivers and facilitators of stigma are needed to inform appropriate interventions in a given context. Systematically collected information regarding the manifestations of stigma is required for researchers and program evaluators to assess the impact of interventions to reduce stigma or mitigate the related harmful consequences. Such information is also important for health facility administrators to identify when training or changes to institutional policies are required to ensure a stigma-free healthcare environment. Affected communities and advocates can use information on stigmatizing practices, as well as the experiences and realities of affected individuals, to raise awareness among the general population and policy-makers to facilitate change. At the national level, data on the outcomes of stigma for affected populations and for organizations and institutions is needed to inform funding for and the scale of programming to address health-related stigma. Such information will also help to identify gaps where new interventions or programs are required.

### Why a new framework and how to use it?

Since sociologist Erving Goffman published his seminal work on stigma in 1963, research on stigma across the disciplines of sociology, psychology, social science, medicine, and public health have expanded, and much is now understood about how stigma operates and induces harm in the context of different diseases and identities. Yet, progress has stalled in our collective ability to tackle stigma and its harmful consequences. Therefore, cross-disciplinary and cross-disease research and collaboration are urgently required to move forward.

The Health Stigma and Discrimination Framework is intended to be a broad, orienting framework, akin to Pearlin’s Stress Process Model, which was developed to *give some conceptual organization to the diverse lines of research that were – and still are – underway* [50]. It is our hope that the framework will enable stigma researchers across disciplines to standardize measures, compare outcomes and build more effective, cross-cutting interventions. In addition, researchers can use the framework to generate research foci, to explore multiple health issues, and consider the interaction between multiple identities, social inequalities and health issues. The framework can also point to areas where clinicians, program implementers, and policy-makers can focus greater attention to better meet the needs of and improve health outcomes among their clients, communities, and societies more broadly. Implementation science approaches can advance how we tailor and apply the framework to guide stigma and discrimination reduction interventions and policies, for example, in defining the target audience for change, what specific drivers and facilitators of stigma should be addressed, what intervention or policy components are appropriate to address them, and how to measure change in specific outcomes overtime.

## Practical applications

To demonstrate the cross-cutting nature of the Health Stigma and Discrimination Framework, we examine how it applies to both communicable and non-communicable health conditions. We review health conditions in roughly chronological order to provide perspective on how health-related stigma has been applied to new and emerging conditions throughout the course of human history. While the different domains of stigma articulated in the framework may not apply in the exact same way across all health conditions, health-related stigmas share a number of commonalities that warrant underscoring.

Firstly, social exclusion rooted in stigma appears to be a response to threat, varying across health-related stigma to the degree to which the source of threat is physical (such as fear of biological contagion, fear of violence and harm) or symbolic (such as aversion based on perceptions that the person does not adhere to central cultural values). Across the various health-related stigmas, people negatively stereotype, display prejudice toward, and discriminate the group and its members, although the content of the stereotype (e.g. being promiscuous, unclean) and the rationalization for the bias differ across the groups. In addition, these conditions differ in the extent to which they are concealable and thus in the way people cope with and manage their stigmatized identity, but all involve anticipated, experienced, and internalized stigma. Finally, how people cope with and manage stigma often adversely affects their health, both in terms of the stress it causes and in the underutilization of services available to them. Table 1 highlights both the commonalties and differences in drivers, facilitators, intersecting stigmas, manifestations, outcomes, and impacts relevant to leprosy, epilepsy, mental health, cancer, HIV, and obesity/overweight, which are further explored below.

**Table 1:** Illustrative examples of how the Health Stigma and Discrimination Framework can be applied to different health conditions

Health condition	Drivers^a^	Facilitators^a^	Intersecting stigmas^a^	Manifestations^a^ (experiences and practices)	Outcomes (affected populations)	Outcomes (organizations and institutions)	Impacts
Leprosy	Fear of contagion, social exclusion, and disfigurement; Beliefs that persons affected by leprosy must have sinned, are ritually impure (Hinduism); have broken taboos (e.g. sexual relations during a woman’s period); belief that leprosy is hereditary	Persons affected by leprosy often have a low SES, have low or no education, low or no awareness of human rights, and are not used to speaking up for themselves	Gender, ethnic background (e.g. caste) in several societies	Experiences: The identity of persons affected is spoiled – they lose status and reputation; this also affects family members They face restrictions in social participation, e.g. problems to find or keep work, problems in accessing education, diminished opportunities for marriage or problems in marriage, problems with friendships, problems in using public facilities, and concealment Practices: Negative attitudes, stereotypes and prejudice towards people with leprosy persist in communities	Concealment may cause delay in treatment, poor treatment adherence, and poor treatment outcomes	Working in leprosy services is unpopular and thus good, well-qualified staff is difficult to find; patients still sent to leprosy hospitals, even for non-leprosy-related conditions, which can lead to poor quality of health services and high turnover of staff	Reduced mental wellbeing, depression and anxiety, (attempted) suicide, aggravated poverty due to loss of income, increased severity of disability, reduced quality of life, prolonged transmission of bacilli in community
Epilepsy	Fears about productivity and longevity (ability to contribute to society)	Religion, supernatural beliefs	Other health conditions (e.g. cerebral palsy), gender, race	Experiences: Employment discrimination, internalization of stigma Practices: Social rejection and distancing, stereotypes about people with epilepsy and their ability to be productive members of society	Treatment self-efficacy, medication adherence	Employment and driving restrictions	Quality of life
Mental health	Beliefs that persons with mental health issues are dangerous (unpredictable, violent), responsible for their issue, cannot be controlled or recover, should be ashamed	Persons with mental health issues viewed as incompetent (cannot work or live independently) or may not be empowered to claim their rights	Race, gender, sexual orientation	Experiences: Internalized stigma, perceived stigma, experienced stigma, discrimination, secondary stigma Practices: Persistent negative public attitudes, opinions and intentions, for example, regarding having a person with mental health issues provide childcare, teach children, marry into the family, attempt self-harm, or hold authority positions	Delays people from accessing, engaging in, and completing mental health treatment	Enactment of protective laws and policies at the national and state-levels and in workplaces, including health facilities	Lowered self-efficacy and self-esteem, compromised engagement in employment and independent living, depression, poor quality of life
Cancer	Fear of infection, perceptions of disfigurement, attributions of blame for contracting the disease, guilt, shame and blame	Religion and culture, perceived responsibility and controllability of cause	Smoker, obesity	Experiences: Internalization of stigma Practices: Social rejection, avoidance, distancing	Delayed screening and treatment seeking, disruption of personal relationships, financial burden	Employment and driving restrictions, health insurance coverage	Quality of life, motivation and efforts to conceal condition, morbidity and mortality
HIV	Fear of infection, fear of economic ramifications due to chronic nature of health condition, fear of poor productivity and longevity, social norm enforcement	Laws criminalizing HIV infection, unenforced protective laws regarding key populations (i.e. men who have sex with men, sex workers, injection drug users, etc.), the availability of universal protection supplies in health facilities, prevailing norms about populations most vulnerable to HIV infection	Sexual orientation, occupation (i.e. sex work), race, substance use	Experiences: Social rejection and distancing, gossip, poor healthcare, internalization of stigma, secondary stigma for family and healthcare workers providing care to people living with HIV Practices: Discriminatory attitudes about people living with HIV, stereotypes and prejudice	HIV risk behaviors, HIV testing, engagement and retention in care, initiation and adherence to medication	HIV-related laws and policies (i.e. criminalization of transmission, travel restrictions), workplace policies, pre- and in-service training curricula for healthcare providers, and other duty bearers	HIV incidence, morbidity and mortality, social inclusion, quality of life
Obesity and body weight	Beliefs that body weight is controllable and people are responsible for their obesity or overweight; Association with laziness and irresponsibility, which violates basic tenets of the Protestant work ethic; Perceived as an atypical physical feature, aversion may reflect the ‘psychological immune system’	Discrimination based on weight not prohibited by federal law in the US, seen as violation of cultural norms	Race, gender, ethnicity	Experiences: Internalization of stigma, experience of weight-based teasing among children, adversely affects new dating opportunities and relationships, discrimination in employment, wages and promotions, environmental stigma (environmental cues, such as size of airline seats and hospital beds) that makes non-normative weight highly salient Practices: Social rejection, distancing, biases within healthcare, media presentations of ideals in health and beauty, as well as portraying overweight as an undesirable characteristic	Vulnerability to depression, low self-esteem, poor body image and maladaptive eating, avoidance of physical exercise, strong experiences of anticipated and perceived stigma	Some evidence of under-utilization of healthcare resources, delay and avoidance of preventive care, one state (Michigan) and some cities (e.g. San Francisco, CA and Binghamton, NY) have laws prohibiting discrimination based on weight, limited effectiveness of interventions to reduce weight-based stigma and discrimination	Increased susceptibility to type 2 diabetes and some evidence of threat to cardiovascular health, quality of life

^a^The examples of drivers, facilitators, intersecting stigmas and manifestations provided in the table are intended to be illustrative. Researchers, clinicians, program implementers, and policy-makers would ascertain the most relevant aspects of each of these domains in their context, or with the specific population they are working with, to apply the framework in support of stigma and discrimination research and reduction efforts

### Leprosy

Leprosy is perhaps the oldest stigmatized health condition known to humankind [51]. Most major religious scriptures make mention of leprosy, often as a condition to be avoided and/or as a divine supernatural punishment for sin or breaking a taboo [52]. The notion that leprosy – or a group of skin diseases that included leprosy – was contagious was already present in the Old Testament of the Bible. Fear of contagion and social exclusion remains closely tied to the image of leprosy [53–55] and the belief that leprosy is hereditary is also widespread [54, 56]. Together, these factors drive the stigmatization process for people living with leprosy.

The fact that persons affected by leprosy often have a low socioeconomic status, a low level of education and little awareness of human rights increases people’s vulnerability to discrimination [57]. In South Asia, a low-caste background can add a further, intersecting layer of stigma, as is the case for women in many endemic countries [58]. The stigma attached to leprosy typically manifests as a ‘spoiled identity’ in the affected person, affecting status and reputation, including that of family members [54, 59]. Social participation may be severely restricted, including problems in finding or maintaining a job, reduced access to education, reduced opportunities in finding a marital partner or problems in ongoing marriages, and sexual health [52, 60–62].

Further, many persons affected seek to conceal their condition [63, 64]. Concealment causes stress and anxiety, but may also lead to a delay in presenting for diagnosis and treatment [65, 66]. When treatment is delayed, the severity of disability may increase [67, 68]. Others may opt to discontinue treatment rather than risk ‘being found out’ [64]. At the personal level, these outcomes of stigma lead to a number of negative impacts for people living with leprosy, such as reduced quality of life and mental wellbeing, including a much increased risk of anxiety and depression [69, 70]. At the organizational level, leprosy-related stigma outcomes may include poor quality of health services and increased staff turnover. At the societal level, the combined impact of these outcomes may be prolonged transmission of bacilli in the community.

### Epilepsy

Epilepsy is a neurological condition characterized by chronic or recurrent seizures. Seizures can lead to individuals crying out, collapsing, bleeding or foaming from the mouth, and losing control of urine and/or stools, and can therefore be frightening to those experiencing or witnessing them. Epilepsy is both concealable and unpredictable – it may be impossible to know that someone has epilepsy until they experience a seizure and it may be impossible to predict the onset of a seizure. Epilepsy-related stigma is largely driven by concerns about productivity and longevity, and fear of infection. Members of the general public endorse beliefs that people with epilepsy cannot contribute meaningfully to society and are poor prospects for marriage and employment [71–73]. Moreover, despite epilepsy not being contagious, some believe that epilepsy is contagious through saliva [74]. Such fears of contagion may be particularly problematic when they are endorsed by first responders, including police officers [75].

Religious and supernatural beliefs act as facilitators of epilepsy-related stigma in some contexts, with some believing that epilepsy is a curse or caused by witchcraft [76]. Risk factors for epilepsy include other health issues (e.g. cerebral palsy, birth asphyxia, stroke) and injuries (e.g. traumatic brain injury), and therefore epilepsy-related stigma may intersect with these other health-related stigmas. People with epilepsy experience a number of manifestations, such as social rejection and exclusion in a range of contexts, including familial and romantic [77]. Children with epilepsy have lower educational achievement and adults with epilepsy experience discrimination within the workplace [76]. Adults with uncontrolled seizures are less likely to be employed and more likely to report job problems when employed [77]. Outcomes of epilepsy-related stigma include lower self-efficacy surrounding treatment engagement and lower medication adherence [4]. Institutional outcomes include stigmatizing policies such as driving and/or employment restrictions that may be disproportionate to illness severity [78]. Epilepsy-related stigma ultimately undermines the quality of life of people living with epilepsy [72].

### Mental health

Mental health-related stigma is often grounded in stereotypes that persons with mental health issues are dangerous (unpredictable, violent), responsible for their mental health issue, cannot be controlled nor recover, and should be ashamed [79]. Persons with mental health issues are often viewed as incompetent and unable to work or live independently [79]. Negative public attitudes, opinions, and intentions persist and are reported across diverse global contexts [80–83]. For instance, findings from the Stigma in Global Context – Mental Health Study, examining responses to scenarios of depression and schizophrenia in 16 countries [84], indicated that core ‘backbone’ stigmatizing beliefs remain across settings with regards to having a person with mental health issues provide childcare, teach children, marry into the family, attempt self-harm, or hold authority positions.

Race and gender appear to intersect with mental health-related stigma, influencing its severity. For example, a higher risk for psychiatric disorders among Caribbean-born versus US-born black men has been reported [85] and greater embarrassment in seeking mental health care has been reported among Somalian-born participants compared to US-born black participants [86]. Certain mental health concerns are perceived as masculine (e.g. addiction, antisocial personality disorder) and others as feminine (e.g. eating disorder), and public stigma towards issues perceived as masculine appears to be higher than towards those perceived as feminine [87, 88]. There are also gender differences in perceived stigma, where men may experience elevated stress regarding disclosing mental health issues in comparison to women [89]. Anticipated and perceived stigma are common manifestations of mental health-related stigma, contributing to fear of acknowledging one’s mental health issue and possibly leading to shame and avoidance regarding seeking mental health care [90, 91]. Mental health-related stigma also has a profound influence on life opportunities and persons realizing their goals and potential; it is associated with lower self-efficacy and self-esteem and compromised engagement in employment and independent living [92].

Public policy responses in some countries have gone a long way towards reducing or ameliorating the harmful effects of mental health-related stigma at the organizational and institutional levels. For example, in the US, the Americans with Disabilities Act [93] enacted in 1990 called for preventing discrimination on the basis of mental health and for the social inclusion and participation of persons with mental health issues in society. In 1999, this was followed by Mental Health: A Report of the Surgeon General [94] to inform the public of mental health issues and raise awareness of stigma and discrimination. Additionally, California’s Mental Health Services Act in 2004 [95] addressed stigma at institutional, societal and individual levels, including social marketing, training, and a focus on cultural competence.

### Cancer

Cancer encompasses a large group of diseases characterized by the uncontrolled growth and spread of abnormal cells. Despite the fact that many cancers can be cured or at least effectively controlled, it remains a highly stigmatized condition, with some types of cancer more stigmatized than others [96]. One key factor in the stigmatization of different types of cancer involves perceptions of the individual’s responsibility for having the disease. For example, cancers of the lung are highly stigmatized [1] due to the belief that smoking is their primary cause, which is believed to be under the person’s control [97]. Most people have negative explicit and implicit attitudes toward smoking and those who smoke [98], which may further strengthen the stigmatization of people with lung cancer. A second factor underlying cancer-related stigma is the degree to which the disease causes apparent disfigurement such as cancers of the throat or mouth. As with other physical conditions, such as weight loss/gain or leprosy, the physical abnormalities associated with some forms of cancer activate the behavioral immune system, eliciting negative emotions such as disgust or aversion, distancing, and avoidance [99].

The experience of cancer-related stigma has important psychological, physical, and social consequences. Psychologically, it is associated with depression, anxiety, and demoralization among patients with cancer [100]. Individuals who experience greater cancer-related stigma tend to delay more in seeking medical care [101] and often attempt to conceal their disease from others [102]. To the extent to which people experience stigma and shame associated with their disease, such as is common with people with lung cancer, they often experience disruption in their personal relationships and decreased marital satisfaction, as well as increased depression, particularly when they blame themselves for their illness [103]. Greater internalization of cancer-related stigma leads to lower self-esteem and poorer mental health, smaller social networks and less opportunity to receive social support, and greater anticipated social rejection, all of which compromise the quality of life [104].

The stigma associated with cancer varies across religions and related cultures. Although women who are members of ultra-Orthodox Jewish communities are at heightened risk for both breast and ovarian cancer due to an increased probability of being carriers of certain genes associated with these cancers given their Eastern and Central European ancestry, they tend to have low screening rates, low health literacy, and poor health practices because of the stigmatization of cancer in these communities [105]. Fears that a diagnosis of breast cancer will dim prospects for arranged marriages have been shown to discourage single Muslim women from accessing treatment for breast cancer in Pakistan [106]. Similarly, South Asian immigrant women of many different faiths in Canada share the belief that having a breast cancer diagnosis would threaten a family’s social status and lead to spousal rejection [106].

### HIV

HIV is a potentially life-threatening disease caused by a virus that weakens the immune system and spreads through blood and sexual contact. HIV-related stigma is driven by several factors, including (1) fear of infection, where people living with HIV (PLHIV) may be perceived as threatening due to the infectious nature of HIV; (2) concerns about productivity and longevity, where PLHIV may be perceived as poor prospects for employment, friendships, and romantic relationships; and (3) social norm enforcement, since HIV risk is related to a range of socially stigmatized behaviors (e.g. same-sex sexual relations, injection drug use, sex work) and therefore PLHIV are devalued due to their perceived associations with these behaviors [107, 108]. Factors that facilitate HIV stigma range from laws that criminalize HIV transmission or specific professions (e.g. sex work) or behaviors (e.g. same-sex sexual relations, injection drug use) to the lack of universal protection supplies in health facilities. Key populations for HIV include men who have sex with men, people with histories of injection drug use, racial and ethnic minorities, and sex workers, and therefore stigmas that intersect with HIV include those associated with sexual orientation, substance use, race, and occupation [36, 109].

PLHIV, including adolescents and young people, report a range of stigmatizing experiences from others, including social rejection, exclusion, gossip, and poor healthcare, and are at risk of internalizing stigma [110]. The level of HIV stigma in communities and societies influences a number of stigma practices, such as discriminatory attitudes among the general public and healthcare workers, and harmful stereotypes and prejudices that can lead to stigmatizing behavior towards PLHIV (exclusion, verbal abuse, etc.). Outcomes of HIV stigma for people at risk of or living with HIV include engagement in greater HIV risk behaviors, lower rates of HIV testing, worse engagement and retention in HIV care, and worse initiation and adherence to medication [3, 44, 111]. Institutional outcomes include stigmatizing policies such as those that criminalize PLHIV who do not disclose their HIV status to their partners or prohibit PLHIV from traveling. Finally, HIV-related stigma has downstream effects on HIV incidence as well as morbidity, mortality, and quality of life for PLHIV [3, 109].

### Overweight and obesity

The stigma associated with weight is particularly strong, pervasive, and openly expressed. There seem to be minimal social norms prohibiting weight shaming, making it particularly problematic. It develops relatively early in socialization, emerging as early as 31 months [112]. Obesity and overweight are often perceived as culturally non-normative, and therefore people with obesity or overweight are often perceived unfavorably, negatively stereotyped, and discriminated against. Additionally, since weight is generally perceived as personally controllable, overweight implies negative personal qualities. Individuals with obesity are often blamed for their weight status and stereotyped as lazy, lacking willpower, incompetent, and unattractive, particularly in cultures that hold core values, such as the Protestant Work Ethic, that emphasize self-control and hard work [113]. In addition to concerns about character, because obesity and overweight are perceived as abnormal physical features, they may activate the behavioral immune system [99] and elicit disgust and related concerns about disease avoidance [114], which leads to distancing and other direct forms of social rejection. Weight-based disparities are well documented in employment, healthcare, education, and interpersonal outcomes [115, 116].

Experiencing and anticipating weight-based stigma (including discrimination, teasing and bullying, social rejection, and other forms of unfair treatment) adversely affects the mental and physical health of people with overweight or obesity [117]. Psychologically, experiencing greater weight-based discrimination is associated with heightened distress (including depression and anxiety) and low self-esteem generally, as well as demoralization and diminished confidence in being able to pursue health-promoting behaviors. Physically, people who experience greater weight-based stigma display less cardiovascular fitness, muscular strength, and endurance [118]. Further, since exposure to weight-based stigma generally reduces motivation, intentions, and feelings of efficacy related to engaging in health-promoting behaviors, weight-based stigma has adverse effects on weight management. Consequently, experiencing more weight-based stigmatization predicts greater caloric consumption and reduced energy expenditure during weight-loss treatment [119]. Thus, weight stigma may contribute to obesity-related health problems due to added stress and reduced engagement in health-promoting behaviors, which jointly operate to increase or maintain excess weight.

In healthcare settings, women who perceive stigmatization from their providers report delaying use of preventive health services for fear of being judged or embarrassed [120]. This avoidance of care allows for untreated problems to progress to a more advanced stage that may be more difficult to treat, thus exacerbating health problems. Moreover, these psychological, physical, motivational, and behavioral effects of weight-based stigma are particularly strong among individuals who internalize this stigma to a greater degree. In terms of responses at the public policy level, there are currently no federal laws against weight-based discrimination; however, one state (Michigan), and a limited number of cities in the US, legally prohibit weight-based discrimination.

## Discussion

The Health Stigma and Discrimination Framework provides an innovative and alternative method to conceptualize and respond to health-related stigmas. Applicable across a range of health conditions and diseases, the framework highlights the domains and pathways common across health-related stigmas and suggests key areas for research, intervention, monitoring, and policy. This crosscutting approach will support a more efficient and effective response to addressing a significant source of poor health outcomes globally.

The Health Stigma and Discrimination Framework has practical applications for program implementers, policy-makers, and researchers alike, providing a ‘common ground’ to inform discourse around research priorities, developing innovative responses and implementing them at scale. For program implementers, the framework can inform the combination and level of interventions most appropriate for responding to a specific type of health-related stigma. For policy-makers, the framework has the potential to lead to efficiencies in funding for and implementation of efforts to reduce health-related stigmas. Lastly, for researchers, the framework should enable more concise and comparable measures of stigma that can be compared across health conditions and diseases by removing the disease siloes of the past and replacing them with common domains and terminology that is more accessible. The framework should also enable crosscutting research endeavors to develop and test interventions that more appropriately address the lived realities of vulnerable populations accessing healthcare systems.

People are not defined by just one disease or one perceived difference, they have complex realities in which to maneuver in order to protect their health and wellbeing, and public health interventions must be responsive to these realities.
